# Supplementary material for: From sequence to enzyme mechanism using multi-label machine learning
Source: BMC Bioinformatics. 2014 May 19;15:150. doi: 10.1186/1471-2105-15-150 (PMC4229970; doi:10.1186/1471-2105-15-150)
Supplement: Additional file 2 — Java code of ml2db. Additional file ml2db_code.tar.gz contains the Java source code to run the multi-label machine learning experiments and save the results to database. The code’s Javadoc is included. [file 1471-2105-15-150-S2.zip › additional file 2/ml2db/ml2db/doc/main/ml2db/weka/InstancesFiller.html]

InstancesFiller


JavaScript is disabled on your browser.


- Overview
- Package
- Class
- Use
- Tree
- Deprecated
- Index
- Help

- Prev Class
- Next Class

- Frames
- No Frames

- All Classes

- Summary:
- Nested |
- Field |
- Constr |
- Method

- Detail:
- Field |
- Constr |
- Method


main.ml2db.weka

## Class InstancesFiller

- java.lang.Object
- - main.ml2db.weka.InstancesFiller

- Direct Known Subclasses:
  :   MulanInstancesFiller

  ---

    

  ```
  public class InstancesFiller
  extends java.lang.Object
  ```

  Generates and fills individual instances for a weka data set.

  Version:
  :   21 Feb 2008

  Author:
  :   Luna De Ferrari ldf(at)st-andrews.ac.uk

- - ### Constructor Summary

    Constructors

    Constructor and Description

    `InstancesFiller(DataSetGenerator dataSetGenerator)`
  - ### Method Summary

    Methods

    | Modifier and Type | Method and Description |
    | --- | --- |
    | `weka.core.SparseInstance` | `fillInstance(java.lang.String thisInstanceName, weka.core.SparseInstance instance)` Returns an instance filled with all its attribute values. |
    | `DataSetGenerator` | `getDataSetGenerator()` |

    - ### Methods inherited from class java.lang.Object

      `equals, getClass, hashCode, notify, notifyAll, toString, wait, wait, wait`

- - ### Constructor Detail


    - #### InstancesFiller

      ```
      public InstancesFiller(DataSetGenerator dataSetGenerator)
      ```
  - ### Method Detail


    - #### fillInstance

      ```
      public weka.core.SparseInstance fillInstance(java.lang.String thisInstanceName,
                                          weka.core.SparseInstance instance)
      ```

      Returns an instance filled with all its attribute values.

      Parameters:
      :   `thisInstanceName` - instance name
      :   `instance` - a weka instance

      Returns:
      :   a filled instance


    - #### getDataSetGenerator

      ```
      public DataSetGenerator getDataSetGenerator()
      ```


- Overview
- Package
- Class
- Use
- Tree
- Deprecated
- Index
- Help

- Prev Class
- Next Class

- Frames
- No Frames

- All Classes

- Summary:
- Nested |
- Field |
- Constr |
- Method

- Detail:
- Field |
- Constr |
- Method
